# Correlation between visual acuity and human leukocyte antigen DRB1*04 in patients with Vogt-Koyanagi-Harada disease

**DOI:** 10.1186/s12886-019-1227-6

**Published:** 2019-11-07

**Authors:** Norihiko Misawa, Mizuki Tagami, Takeya Kohno, Shigeru Honda

**Affiliations:** Department of Ophthalmology and Visual Science, Graduate School of Medicine, Osaka City University, 1-5-7 Asahimachi, Abeno-ku, Osaka-shi, 545-8586 Japan

**Keywords:** Vogt-Koyanagi-Harada disease, HLA-DRB1*04, Japanese patients

## Abstract

**Background:**

The common presence of human leukocyte antigen (HLA)-DRB1*04 in Vogt-Koyanagi-Harada (VKH) disease is well known. The aim of this study was to investigate the relationship between visual prognosis and HLA-DRB1*04 alleles during systemic corticosteroid therapy in patients with VKH disease.

**Methods:**

This retrospective case series included 57 eyes from 29 consecutive patients with treatment-naïve VKH disease who received systemic corticosteroid therapy. Visual acuity, sex, refractive error, central retinal thickness (CRT), central choroidal thickness (CCT), and duration from onset to treatment were measured at initial and final visits. Mean values of parameters were compared with each visit. Genotyping was performed by polymerase chain reaction amplification with sequence-specific primer.

**Results:**

Linear regression showed significant differences in logMAR best-corrected visual acuity between the three groups of homozygotes, heterozygotes, and normal subjects at baseline (*p* < 0.01), at 3 months after treatment (*p* < 0.01). There was no significant differences at 6 months after treatment (*p* = 0.257). No significant differences were detected between the three groups in age, sex, refractive error, CRT, CCT, or duration from onset to treatment.

**Conclusion:**

Alleles of HLA-DRB1*04 might affect visual prognosis and be related to early response after initiation of treatment in VKH disease.

## Background

Vogt-Koyanagi-Harada (VKH) disease is a systemic disorder that is considered to represent an autoimmune disease against melanocytes [1]. In the eye, the disease presents as acute bilateral granulomatous panuveitis, which responds to systemic corticosteroid therapy and generally shows good visual prognosis with relatively short follow-up [2]. A role of genetic factors such as HLA alleles in the development of VKH was first considered in 1976, and was supported by the simultaneous development of VKH in monozygotic twins [3]. The human leukocyte antigen (HLA) system is the locus of genes that encode for major histocompatibility complex (MHC), representing a set of cell surface molecules mediating the interaction of leukocytes [4]. HLA, and HLA-DRB1*04 in particular, therefore plays a key role in immune systemic function, as well as in the pathogenesis of autoimmune diseases, including VKH [5]. A Korean study found that the HLA DRB1*0405 allele conferred an increased relative risk of developing VKH compared with the general population, and that the HLA DRB1*0405-DQA1*0302-DQB1*0401 haplotype was associated with poorer visual prognosis [6]. On the other hand, despite systemic corticosteroid approach, VKH patients often prove refractory to systemic corticosteroid therapy with long follow-up [7, 8]. For predicting prognosis and refractoriness to treatment, the focus in recent years has transitioned from the identification of HLA genes associated with increased risk of VKH to the identification of alternate genes [9, 10]. However, in Japan, HLA has traditionally been examined for uveitis, and accumulated data are available for our facilities. For that reason, we report an investigation of correlations between HLA04 allele type and visual outcomes before and after steroid treatment in the real world.

## Methods

This retrospective case series included 57 eyes from 29 consecutive patients with treatment-naïve VKH disease who visited the ophthalmology department at Osaka City University Hospital between December 2009 and January 2019 and were followed up for more than 6 months after the start of systemic corticosteroid therapy. VKH disease was diagnosed according to the criteria of Sugiura [11] and the VKH Disease Committee [12]. None of the patients had any medical or ocular history at the initial visit. Changes in central retinal thickness (CRT) and central choroidal thickness (CCT) were assessed by vertically and horizontally oriented enhanced depth imaging optical coherence tomography (EDI-OCT) of the macula (Spectralis HRA + OCT Heidelberg Engineering, Heidelberg, Germany) for up to 3 months after treatment. Measurements of CRT and CCT in each eye were then reconfirmed by three experts (NM, MT, and SH) by checking the OCT images. Patients received corticosteroid regimens of pulse therapy according to the timing of their first visit. Recurrences were defined as the recurrence of anterior chamber cells and/or posterior segment lesions detected by ophthalmic examinations.

In HLA typing by polymerase chain reaction amplification with sequence-specific primers (PCR-SSP), typing specificity is part of the amplification step [9].

The Kruskal-Wallis test and Fisher’s exact test were used to analyze and compare baseline patient characteristics. The relationship between HLA04 allele and visual outcomes was determined using univariate linear regression analysis. Analysis of covariance was used to analyze final visual acuity to clarify the influence of baseline visual acuity. In addition, in the case of regression analysis, an explanatory variable that roughly divided the number of cases by 15 was considered appropriate [13].

Statistical analyses were performed using SPSS Statistics version 22 software (IBM Japan, Tokyo, Japan). Values of *P* < 0.05 were considered statistically significant.

## Results

### Treatment and patient characteristics

Patients received regimens of corticosteroid pulse therapy according to the timing of their first visit. In the 27 patients administered intravenous methylprednisolone, the dose was 1000 mg/day for 3 consecutive days followed by tapering of oral prednisolone (i.e., pulse therapy), as described previously [14]. Oral prednisolone was temporarily increased or restarted in the event of anterior or posterior recurrence of VKH disease. The remaining two patients were administered intravenous prednisolone and tapered from 100 mg/day (i.e., high-dose therapy), as described previously [7]. Oral cyclosporine was not administered in the present study. We calculated duration from onset to steroid administration and duration from first visit to steroid administration in all patients. Mean duration from onset to steroid administration was 17.4 ± 10.7 days and mean duration from first visit to steroid administration was 4.1 ± 3.2 days. Baseline patient characteristics are summarized in Table 1. The mean follow-up was 22 ± 20 months (range 6–105 months). Recurrences of inflammation were observed in 5 patients (17%) over follow-up, and they consisted of visible posterior segment inflammation (subretinal fluid or choroidal white lesions) in all patients.

**Table 1:** Pre-treatment parameters baseline patient characteristics

HLA-DRB1*04 allele type	HLA-DRB1*04 −/−	HLA-DRB1*04 +/−	HLA-DRB1*04 +/+	*p*
Age (years)	47.83 ± 23.78	50.73 ± 17.53	49.52 ± 14.24	0.793*
Sex (male:female)	3:3	9:5	3:5	0.47**
Refractive error (diopters)	−0.93 ± 17.15	−3.31 ± 2.99	−2.23 ± 2.74	0.81*
CRT (μm)	489.4 ± 258.4	557.5 ± 197.5	517.5 ± 190.5	0.86*
CCT (μm)	210 ± 297	310 ± 317	518 ± 191	0.86*
Duration from onset to treatment (days)	18.7 ± 12.68	16.07 ± 11.20	19.25 ± 9.48	0.58*

*CRT* central retinal thickness, *CCT* central choroidal thickness

* Kruskal-Wallis test

** Fisher’s exact test

### Visual outcomes

In visual acuity, mean logMAR best-corrected visual acuity (BCVA) values at baseline, at 3 months after treatment and, at 6 months after treatment were 0.34 ± 0.58, 0.01 ± 0.25,and 0.01 ± 0.38 in HLA-DRB1*04 −/−, − 0.16 ± 0.33, − 0.11 ± 0.06,and − 0.06 ± 0.14 in HLA-DRB1*04 +/−, and 0.008 ± 0.14, − 0.13 ± 0.05, and − 0.09 ± 0.24 in HLA-DRB1*04 +/+, indicating significant visual improvements from baseline after treatment in HLA-DRB1*04 +/− and HLA-DRB1*04 +/+group (3 M: *p* < 0.01, *p* < 0.01, *p* = 0.056, respectively) (6 M:*p*=,0.24, *p* < 0.01, *p* = 0.07,respectively). In covariate analysis considering the influence of baseline visual acuity, the HLA-DRB1*04 −/− and HLA-DRB1*04 +/− groups showed significant differences in the final visit (*p* < 0.01). Mean CCT values pretreatment and at final visit after treatment were 489.4 ± 258.4 and 285.2 ± 159.4 μm in HLA-DRB1*04 −/−, 557.5 ± 197.5 and 356.1 ± 135.5 μm in HLA-DRB1*04 +/−, and 517.5 ± 190.5 and 375.8 ± 211.2 μm in HLA-DRB1*04 +/+, indicating changes compared with baseline (*p* = 0.110, *p* < 0.01, *p* = 0.173, respectively). In the HLA-DRB1*04 +/− group alone, mean post-treatment CCT values were significantly decreased compared with baseline CCT.

### Correlation with HLA-DRB1*04 allele

**Fig. 1:**
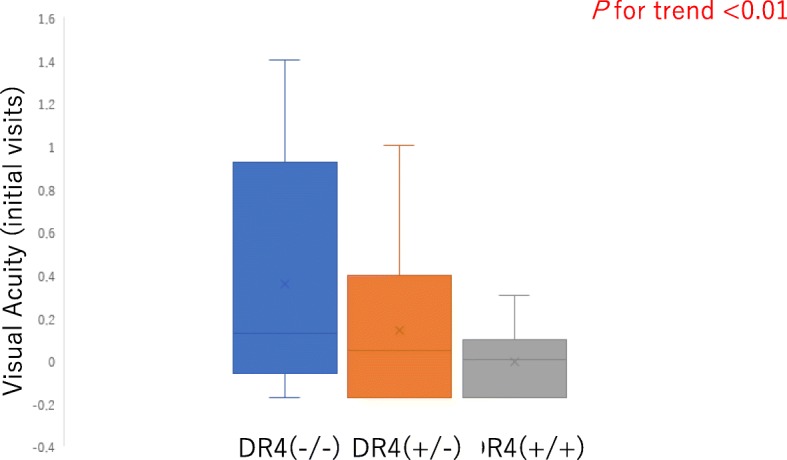
Linear regression for best-corrected visual acuity (BCVA) at baseline. LogMAR BCVA for Vogt-Koyanagi-Harada (VKH) disease at baseline on linear regression among the three groups of homozygotes (HLA-DRB1*04 +/+), heterozygotes (HLA-DRB1*04 +/−), and normal subjects (HLA-DRB1*04 −/−)

HLA-DRB1*04 typing was performed for 5 patients (10 eyes) in normal subjects (HLA-DRB1*04 −/−), 15 patients (29 eyes) in heterozygotes (HLA-DRB1*04 +/−), and 6 patients (12 eyes) in homozygotes (HLA-DRB1*04 +/+). Linear regression analysis showed significant differences among the three groups of homozygotes, heterozygotes, and normal subjects in logMAR BCVA at baseline in Fig. 1 (*p* < 0.01).

**Fig. 2:**
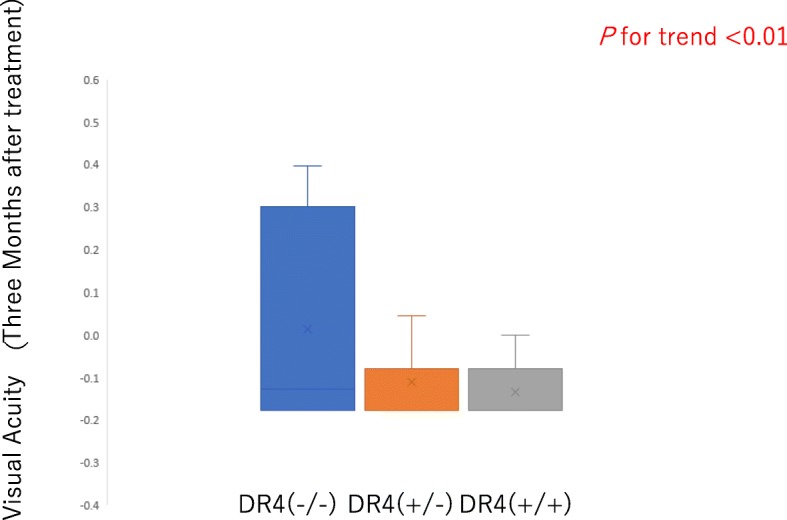
Linear regression in best-corrected visual acuity (BCVA) at final visit. LogMAR BCVA for Vogt-Koyanagi-Harada (VKH) disease at 3 months (after treatment) on linear regression among the three groups of homozygotes (HLA-DRB1*04 +/+), heterozygotes (HLA-DRB1*04 +/−), and normal subjects (HLA-DRB1*04 −/−)

Comparisons of clinical parameters at post-treatment in the three groups are summarized in Table 2. Linear regression analysis found significant differences among the three groups of homozygotes, heterozygotes, and normal subjects in logMAR BCVA at 3 months after treatment in Fig. 2 (*p* < 0.01). There was no significant differences at 6 months after treatment (*p* = 0.25). This result was consistent with the finding of no difference among three groups with regard to choice of treatment regimen according to the period during which the patient visited the clinic (i.e., large dose, earlier; pulse, later). Second, the number of recurrences during follow-up did not differ between the three groups.

**Table 2:** Clinical parameters compared post-treatment among the three groups

	Patient	Duration from onset to treatment(days)	Duration from first visit to treatment(days)	Recurrence	Post-treatment ocular complications	Initital treatment	Final logMAR BCVA R	Final logMAR BCVA L
HLA-DRB1*04 +/+	9	37	11	none	none	paluse	−0.176091259	− 0.176091259
	11	23	2	none	none	paluse	−0.176091259	0
	13	14	1	PSL increased	none	STTA(40 mg),paluse	−0.079181246	−0.176091259
	16	11	9	none	none	paluse	−0.176091259	− 0.176091259
	20	12	1	none	none	paluse	−0.176091259	−0.176091259
	23	25	2	none	Left cataract	paluse	−0.176091259	−0.176091259
	25	9	4	none	none	paluse	−0.079181246	− 0.079181246
	27	23	8	none	none	paluse	−0.079181246	− 0.079181246
HLA-DRB1*04 +/−	2	7	2	none	none	paluse	0	−0.079181246
	4	14	6	none	none	STTA(40 mg),paluse	−0.176091259	−0.176091259
	5	40	2	PSL increased	Left cataract	paluse	−0.079181246	−0.079181246
	8	19	5	none	none	paluse	−0.176091259	−0.176091259
	10	12	7	none	none	paluse		0.301029996
	12	21	0	PSL increased	none	paluse	0	−0.079181246
	14	39	6	PSL increased	none	paluse	−0.079181246	−0.079181246
	17	6	1	PSL resumed	none	PSL40mg,paluse	0	−0.079181246
	18	11	4	none	none	paluse	−0.176091259	−0.079181246
	19	6	1	none	none	paluse	−0.176091259	0
	21	8	1	none	none	paluse	−0.176091259	−0.176091259
	22	23	1	none	none	paluse	−0.176091259	−0.176091259
	26	3	5	none	none	paluse	−0.176091259	−0.176091259
	28	14	12	none	none	paluse	−0.176091259	−0.176091259
	29	18	6	none	none	paluse	−0.176091259	−0.176091259
HLA-DRB1*04 −/−	1	16	2	none	none	paluse	−0.079181246	−0.079181246
	3	13	7	none	Both cataract	palse	0	0.15490196
	6	17	6	none	none	STTA(40 mg),paluse	1	0.301029996
	7	20	1	none	none	paluse	−0.176091259	0
	15	4	4	none	none	PSL30mg	−0.176091259	−0.176091259
	24	42	4	none	none	paluse	−0.176091259	−0.176091259
*P*-value		0.586*	0.87*				0.645*	0.301*

*STTA* sub-tenon triamcinolone acetonide

In terms of visual prognosis of the 57 eyes examined, we documented mild to moderate cataracts in 4 eyes and drug-controllable glaucoma in 3 eyes after treatment, none of which required surgery during follow-up. No differences in the frequency of these ocular complications were seen among patients.

## Discussion

The present study revealed a correlation between HLA04 allele and visual outcomes before and after initiating systemic corticosteroid therapy in treatment-naïve patients with VKH disease. In this study, linear regression showed significant differences in logMAR BCVA at baseline and at 3 months after treatment between the three groups of homozygotes, heterozygotes, and normal subjects. There were no significant differences at 6 months after treatment. We can guess that Alleles of HLA-DRB1*04 might affect visual prognosis and be related to early response after initiation of treatment in VKH disease. From these results. Also, although the statistical study did not yield significant results, we speculate that CRT and CCT might show significant differences with greater numbers of cases of homozygotes, heterozygotes, and normal subjects. These thicknesses may be associated with the final prognosis of the disease [14]. The duration from first visit to steroid administration was very short. This was taken as a likely reason for the low recurrence rate for VKH compared with other reports without the use of immunosuppression therapy [7]. Cyclosporine was approved in 2012, and Adalimumab was also approved by the government for several years. But not all recommended approaches are government approved in Japan. For these reasons, steroid-centered treatment has always been used. But future treatments for recurrent VKH may be changed [15].

Previous studies have reported that HLAs represent a set of cell surface molecules mediating leukocyte interactions. HLA therefore plays an important role in immune system function as well as in the pathogenesis of autoimmune diseases, including VKH. Almost 40 years ago, an association between HLA-BW22J and VKH was reported [16]. Since then, more articles have been published regarding the associations of different HLA types to VKH. Among these, most investigations have focused on the HLA-DR4 serotype and its corresponding allele, HLA-DRB1*04 [17]. Shi et al. reported a meta-analysis confirming the association between VKH and HLA-DR4/DRB1*04, finding that the strength of association differed between ethnic groups, and identifying HLA-DRB1*0404, 0405, and 0410 as risk sub-alleles, and 0401 as a protective sub-allele [18, 19].

For groups not having HLA-DR4/DRB1*04, the diagnosis may be not typical for VKH. However, both homozygous and heterozygous groups showed findings above the regression equation line, and were considered to have certain statistical meaning.

In the study of type 1 diabetes in Japanese, disease susceptibility has been found to differ between homozygotes and heterozygotes [20]. Similarly, disease susceptibility to VKH may differ between homozygotes and heterozygotes. Both before and after steroid treatment, homozygotes displayed the best post-treatment visual acuity. Normal subjects (no HLA-DRB1*04 allele) showed the poorest visual acuity after treatment. This indicates that therapeutic response and sensitivity to steroid treatment may depend on allele HLA-DRB1*04. An understanding of the pathogenic conditions that must exist to explain these results is difficult to reach**.** However, previous reports using methods such as haplotype linkage disequilibrium have suggested associations with genes closely related to immunity and inflammation, such as the *IKBL* gene and *TNFA* gene present in HLA class III [21–23].

The HLA-DRB1*04 allele is known to represent a disease-associated gene that is frequently found in VKH, but our study suggested the possibility of disease resistance in association with this allele. On the other hand, another report found that presence of the HLA-DRB1*04 allele was related to the prolongation of VKH in Japanese patients [24].

We know that the HLA-DRB1*04 allele is the key to VKH, but the details remain elusive. According to the previous report, CCT correlates with vision prognosis in VKH [25]. However, in this case series with possible problems in the limited number of cases, the correlation between allele HLA-DRB1*04 and CCT could not be determined. Another limitation is following periods had variations and the bias cannot be denied completely.

## Conclusion

We report that alleles of HLA-DRB1*04 might affect visual prognosis and be related to early response after initiation of treatment in VKH disease. In the future, the complete genetic predisposition of VKH is expected to be elucidated, leading to the development of next-generation treatments and preventive measures. We acknowledge these potential issues, as well as the need for future worldwide studies into the correlation between the HLA-DRB1*04 allele and visual outcomes in VKH.

## Data Availability

The data generated and/or analyzed for this study is available from the corresponding author upon request.
